# Constitutive Expression Differences and Thermal Responses of Stress-Associated Transcripts in Pacific Abalone

**DOI:** 10.3390/biology15201885

**Published:** 2026-10-19

**Authors:** Xuefan Cui, Di Lan, Huaqing Lin, Weilong Yang, Xin Liu, Qiwen Liu, Shushan Nian, Zhaoxian Li, Xinhua Chen, Guilan Di

**Affiliations:** 1 State Key Laboratory of Mariculture Breeding, Key Laboratory of Marine Biotechnology of Fujian Province, College of Marine Sciences, Fujian Agriculture and Forestry University, Fuzhou 350002, China; cuixuefan@fafu.edu.cn (X.C.); m17750898370@163.com (D.L.); 13562625739@163.com (H.L.); ywl12323@gmail.com (W.Y.); 18461083525@163.com (X.L.); 19223537361@163.com (Q.L.); 13385932325@163.com (S.N.); 2 Institute of Hydrobiology, Chinese Academy of Sciences, Wuhan 430072, China; lizhaoxian@ihb.ac.cn; 3 Laboratory for Marine Biology and Biotechnology, Qingdao National Laboratory for Marine Science and Technology, Qingdao 266071, China

**Keywords:** *Haliotis discus hannai*, thermal stress, constitutive expression, population variation, hepatopancreas, heat-shock response, PI3K–AKT–mTOR

## Abstract

We examined basal and exposure-associated transcript abundance in Pacific abalone. *Pi3k*, *Akt*, *mTOR*, *Hsf1*, *Hsp70*, and *Hsp90* were compared across five anatomical samples and between heat-selected and common populations after identical two-week acclimation. Five transcripts were examined during adult exposure to 30 °C for up to 72 h and after a 15 min larval heat shock. Basal groups had three animal samples; population and adult groups had two or three analyzed samples. Larval groups comprised three pools of 500 individuals. Qualitative histology used three animals per condition. Welch tests on log2 values used Holm correction. Selected animals had higher hepatopancreatic *Pi3k*, *Akt*, *mTOR*, and *Hsp70* levels. Hepatopancreatic *Hsf1*/*Hsp70* means were highest at 12 h and *Pi3k*/*Akt* means at 24 h; peak times were not statistically compared. All five transcripts increased within late-veliger samples, without establishing a stage-by-treatment interaction. Histology showed local discontinuities, including in controls. These patterns identify candidate transcript-level indicators. One adult tank per condition, absent time-matched control comparisons, unverified larval culture independence, small samples, normalization uncertainty, population-history confounding, and absent protein endpoints and direct tolerance measurements limit interpretation.

## Simple Summary

High seawater temperatures challenge Pacific abalone farming, but molecular responses vary among organs and populations. We compared six stress-associated messenger RNAs across five anatomical samples and between heat-selected and common populations after two weeks under the same acclimation conditions. We also examined five transcripts in adults during exposure to 30 °C and in larvae after a brief heat shock. Several transcripts were more abundant in the hepatopancreas of the selected population. Adult expression varied over time, and all five transcripts increased within late-veliger samples. The adult experiment used one tank per condition, so its expression profiles cannot separate heating from tank-related or time-related influences. Larval samples were pools of 500 individuals, with three pools per condition and stage; these do not establish replication across independent spawnings. Histological images provided qualitative context from three animals per condition. These observations identify expression patterns for further investigation, rather than validated indicators of thermal resistance.

## 1. Introduction

Temperature influences the performance of Pacific abalone, *Haliotis discus hannai*, through effects on metabolism, cellular protection, and tissue function. Controlled experiments have shown that the magnitude and rate of temperature change alter oxygen consumption, nitrogen excretion, and digestive-gland enzyme activity [1]. Under sustained thermal exposure, changes in adhesion, foot morphology, and *Hsp90* expression accompany temperature-dependent physiological disturbance [2]. These responses make thermal tolerance an important trait for abalone aquaculture, while also showing why a single molecular measurement may not capture an animal’s capacity to withstand high temperature. Acute-stress transcriptomics in Pacific abalone also implicates endoplasmic-reticulum protein processing, including HSP70 and HSP90 [3], whereas a hybrid abalone study identified temperature-dependent transcriptional changes during heating and recovery [4]. These findings make exposure duration and recovery history important when comparing molecular responses.

Thermal performance varies among Pacific abalone populations. Comparisons of northern and southern stocks have identified differences in growth and post-challenge *Hsp70* and *Hsp90* expression [5]. Cardiac-performance-based studies likewise distinguish heat-tolerant and heat-sensitive lines with different transcriptomic responses [6]. Long-term domestication in warmer environments is associated with altered metabolic regulation and energy allocation [7], and population differences extend to transcriptional and DNA-methylation profiles [8]. Genome-wide association analysis has further implicated loci near genes involved in metabolism, ion transport, signaling, and DNA repair [9]. These findings motivate us to examine the molecular state present before heating, as well as the changes induced during a challenge.

Constitutive expression and inducibility address related but distinct questions. A population may maintain greater basal expression of selected protective genes, respond more rapidly after heating, or tolerate stress with a smaller transcriptional disturbance. Elevated expression can therefore indicate preparedness, ongoing physiological demand, or cellular damage, depending on the context. In Pacific abalone, *Hsp70* has been characterized as a thermally responsive transcript [10], while broader profiling has demonstrated tissue-dependent responses among multiple heat-shock-protein families [11]. An earlier quantitative PCR study also documented a transient *Hsp70* response during recovery from heat shock [12]. Comparisons across tissues and time are needed to distinguish persistent basal differences from short-lived inducible responses.

Heat-shock factor 1 (HSF1) provides a link between protein homeostasis and inducible transcription. Experiments in yeast show that chaperone feedback and phosphorylation can regulate Hsf1 output through separable processes [13]. In small abalone, *Haliotis diversicolor*, *Hsf1* knockdown decreases the expression of several heat-responsive genes [14]. In oysters, differences among HSF1 isoforms and heat-shock-gene promoters contribute to variation in thermal responses [15]. These studies justify examining *Hsf1* with *Hsp70* and *Hsp90*, but they also caution against treating *Hsf1* transcript abundance as a direct measure of HSF1 activation.

The phosphoinositide 3-kinase/protein kinase B (PI3K/AKT) pathway and mechanistic target of rapamycin (mTOR) are additional candidates for describing the molecular context of thermal responses. PI3K/AKT-related responses have been linked to hypoxia- and heat-associated immunosuppression in small abalone [16]. In oysters, PI3K/AKT-mediated phosphorylation of an effector caspase influences heat-induced caspase activation [17]. In human cultured cells, mTOR contributes to HSF1 phosphorylation and the proteotoxic stress response [18]. These functional findings establish biological reasons to examine the corresponding transcripts together, without implying that changes in their messenger RNA necessarily reveal pathway activity or regulatory order in Pacific abalone.

Previous abalone studies have identified thermally responsive transcripts and molecular differences associated with independently measured thermal performance [6,7,11]. How a defined transcript panel varies across anatomical sites, populations, and exposure intervals remains less clear. We evaluated three transcript-level hypotheses: (i) basal relative abundance of *Pi3k*, *Akt*, *mTOR*, *Hsf1*, *Hsp70*, and *Hsp90* differs among hepatopancreas, gill, foot muscle, mantle, and intestine; (ii) basal abundance differs between heat-selected and common populations within anatomical sites; and (iii) *Pi3k*, *Akt*, *mTOR*, *Hsf1*, and *Hsp70* abundance differs from adult baseline during exposure to 30 °C and between before- and after-shock samples within larval stages. The corresponding objectives were to map basal profiles, estimate within-site population contrasts, and describe adult temporal and within-stage developmental contrasts. This targeted combination extends earlier studies without testing a causal heat-tolerance mechanism. The bulk samples contain multiple cell types, and population history, geographical origin, and longer-term culture history may contribute to expression differences.

Following the basal population screen, the exposure experiments focused on *Pi3k*, *Akt*, *mTOR*, *Hsf1*, and *Hsp70* to examine the *Hsp70*-centered response in its signaling-associated transcript context. *Hsp90* was retained in the basal and population comparisons but was not included in the exposure panel. This targeted choice does not imply that *Hsp90* is unimportant during heating or establish that *Hsp70* has a stronger role in thermal tolerance.

## 2. Materials and Methods

### 2.1. Animals and Basal Population Comparison

Pacific abalone were obtained from Fujian Dongyu Marine Health Food Co., Ltd., China. Population R had a history of multigeneration high-temperature selection and is termed the heat-selected population; population D was a common cultured stock of northern origin without that selection history. These labels describe provenance, because thermal tolerance was not independently measured in the sampled animals. Healthy, undamaged animals of similar size, with shell lengths of 5–8 cm and body weights of 30–50 g, were used. Before basal sampling, both populations were acclimated for two weeks under the same recirculating-seawater conditions: 20 ± 1 °C; salinity, 30 ± 1; pH 8.0 ± 0.2; and continuous aeration. Fresh kelp was supplied once daily, and residual feed and waste were removed promptly. Feeding and activity were monitored, and sampling followed recovery of normal feeding. Basal comparisons examined hepatopancreas, gill, foot muscle, mantle, and intestine. This common acclimation standardized recent conditions but did not separate selection history from geographical origin or earlier husbandry history.

Animals were rinsed with sterile seawater before dissection. Hepatopancreas and gill samples were separated from surrounding tissues; foot muscle was sampled after removal of the surface epithelium; mantle was sampled at the margin; and intestinal contents were gently removed by rinsing with phosphate-buffered saline. Samples were collected under cold conditions, placed in labeled tubes on dry ice, and stored at −80 °C. An independently sampled animal was the biological unit for each adult expression group. Replicate columns were not paired across groups, and PCR technical reactions were not treated as biological replicates. Foot muscle was the designated comparator for within-gene basal contrasts, providing a common anatomical reference for presentation; this choice does not imply that foot muscle is biologically invariant or functions as a qPCR normalization gene.

### 2.2. Adult Thermal Exposure

The adult time course and histological examination used the common population. A total of 120 healthy, undamaged adults of similar size were acclimated for two weeks under the conditions described in Section 2.1. Sixty animals were randomly allocated to one 500 L control tank and 60 to a second 500 L heat-treatment tank. The control tank remained at 20 ± 1 °C. The heat-treatment tank was warmed from 20 to 30 °C at 2 °C h^−1^, with exposure duration counted from attainment of 30 °C. Animals were observed every 3 h. Water in the heated tank was replaced every 12 h using seawater prewarmed to 30 °C. The 0 h baseline samples came from the 20 °C control tank; exposed animals were sampled at 6, 12, 24, 48, and 72 h after reaching 30 °C. Three animals were sampled per time point, with two or three expression values available for analysis in the specified gene groups (Section 2.6). *Pi3k*, *Akt*, *mTOR*, *Hsf1*, and *Hsp70* were measured in the five anatomical samples. Each condition used only one tank: animals were within-tank biological subsamples, not independent temperature-treatment replicates. Temperature was therefore confounded with tank, and the reported contrasts did not include time-matched control-tank samples beyond baseline.

### 2.3. Heat Shock During Early Development

Developmental samples were collected during the autumn hatchery season. Broodstock comprised five males and 40 females with well-developed gonads, and artificial breeding was followed by continuous observation of development. Samples were assigned to the trochophore, veliger, and late-veliger stages at approximately 12, 16, and 48 h post-fertilization, respectively. Stage terminology and morphological interpretation were guided by published descriptions of abalone larval development [19]; the developmental times in that small-abalone study were not used as fixed thresholds for Pacific abalone. Larvae were transferred with a pipette to 40 µm cell strainers and immersed in seawater at 30 °C for 15 min. Before- and after-shock samples were collected separately within each stage for *Pi3k*, *Akt*, *mTOR*, *Hsf1*, and *Hsp70*. Each sample pooled 500 larvae, with three pools per condition and stage. The pool was the measurement unit; 500 larvae did not constitute 500 replicates. Independent spawning-batch or culture-vessel replication was not specified, so pool-level contrasts do not establish between-batch reproducibility. The brief treatment examined acute transcript responses; no dose-duration optimization, stage-specific tolerance threshold, or larval survival validation is reported.

### 2.4. RNA Preparation and Quantitative PCR

Total RNA was extracted using an RNAsimple Total RNA Kit (DP419; TIANGEN Biotech, Beijing, China). First-strand complementary DNA was synthesized using All-In-One 5X RT MasterMix with gDNA Removal (Applied Biological Materials, Richmond, BC, Canada) and stored at −20 °C. Quantitative PCR was performed on a QuantStudio 5 (Applied Biosystems, Thermo Fisher Scientific, Waltham, MA, USA) using ChamQ Universal SYBR qPCR Master Mix (Vazyme Biotech, Nanjing, China), with *β-actin* as the single normalization gene specified in the original assay protocol. Preliminary specificity tests produced a single sharp melting-curve peak. This supports a dominant amplification product but does not establish amplification efficiency or reference-gene stability. Reference-gene testing in developmental Pacific abalone samples highlights the need to establish stability across the actual stages and treatments [20,21]. Primer sequences are provided in Table S1. Each 20 µL reaction contained 10 µL of 2× master mix, 0.4 µL of each primer at 10 µM, 1 µL of complementary DNA, and nuclease-free water. Cycling comprised 95 °C for 30 s and 40 cycles of 95 °C for 10 s, 60 °C for 20 s, and 72 °C for 20 s, followed by melting-curve acquisition. Each biological sample was assayed in three technical reactions. Relative expression was calculated using the 2^−ΔΔCt^ method [22]. Standard-curve amplification efficiencies and experiment-specific stability estimates for *β-actin* were not reported, leaving equal target/reference efficiencies and stable reference expression unverified. Raw target/reference Ct/Cq, ΔCt, and calibrator records were not available for an independent check of the reported fold changes. Analyses therefore used the recorded positive relative-expression values, without treating values from different gene assays as absolute between-gene abundance.

### 2.5. Histological Examination

Hepatopancreas, gill, intestine, and mantle samples from three control animals at 20 °C and three animals after 24 h at 30 °C were prepared for histology. Tissue pieces were fixed in 4% paraformaldehyde at 4 °C for 24–48 h, dehydrated through graded ethanol, cleared in xylene, embedded in paraffin, and sectioned at 4–5 µm. Ten sections were prepared per tissue per animal, with three to five microscopic fields examined per section. Sections were stained with hematoxylin and eosin. Animals were the biological samples; sections and fields were nested observations. The displayed assessment used one available digitized-slide record per anatomical site and condition as an illustrative source, rather than a quantitative survey of all sections from all six animals. Low-magnification fields provided architectural context, with corresponding high-magnification fields and calibrated scale bars (0.2513854 µm per pixel at the highest image level). The single tank per condition also limits treatment-level interpretation of the histology.

Microscopic descriptions used operational, non-diagnostic criteria: a discontinuity was a visible interruption of a glandular or epithelial profile in the section plane; fragment-like material was material separated from the adjacent continuous profile; and a dense cellular region was a local increase in closely packed hematoxylin-stained profiles. These appearances were not designated necrosis or inflammatory infiltration. Hepatopancreas and gill scans were surveyed across low-magnification fields; illustrative peripheral fields were selected to show local appearances, while additional central/continuous fields document within-section variability. Selection was not blinded, and no lesion score, prevalence estimate, or inferential histological test was calculated. Scale bars indicate image calibration, not biological replication.

### 2.6. Statistical Analysis

Expression experiments were designed with three biological samples per group: adult animal samples or larval pools of 500 individuals. Means ± SD and statistical tests used the recorded measurement values. Five groups had two available measurements: R intestinal *Hsp70*, R gill and intestinal *Hsp90*, and gill *mTOR* at baseline and 6 h; all other groups had three. No missing value was replaced by a group mean or another imputed biological replicate. Adult animals were subsamples within one tank per condition, and independent spawning or culture replication for larval pools was not established. These counts describe measurement-level replication, not independent tanks or spawning batches. Histology used three animals per condition, with sections and fields nested within animals.

Two-sided unpaired Welch *t*-tests were applied to log2-transformed positive relative-expression values for specified simple contrasts: each non-foot sample versus foot muscle within a gene; R versus D within each anatomical site and gene; each adult exposure time versus the 20 °C baseline; and after versus before heat shock within each larval stage and gene. Holm correction was applied across 24 basal, 30 population, 125 adult temporal, and 15 developmental contrasts [23]. The adult comparisons were adjusted as a single family across all five anatomical sites. The 125 adult contrasts are exploratory comparisons of animal samples from two unreplicated tanks; their adjusted *p*-values cannot support independent temperature-treatment inference. Larval contrasts describe the sampled pools, with between-culture independence unverified. Adjusted *p* < 0.05 was used to annotate the defined comparisons, not to establish biological importance or equivalence for nonsignificant results. Fold differences are ratios of geometric means; graphs show arithmetic means and SD. Unpaired tests reflected the absence of paired replicate columns and assumed approximately normal log2-scale errors. No population × anatomical site, anatomical site × time, or stage × treatment interaction was estimated. A factorial reanalysis cannot recover missing independent tank replication. Different significance outcomes do not establish interactions, and consecutive adult time points were not compared. Statistical calculations used custom Python scripts (https://www.python.org) and NumPy (https://numpy.org); both websites were accessed on 19 October 2026.

Spearman coefficients summarize exploratory covariation among the five temporal profiles within each anatomical site across six time positions (Figure S1). The original nominal probabilities were evaluated over all 720 permutations of six ranks and Holm-adjusted across 50 gene pairs. Because these are group means along a time series rather than independent animals, temporal dependence can violate exchangeability. The matrices and probabilities are retained only as exploratory descriptions, not confirmatory evidence for association, absence of regulation, or causal signaling.

We additionally assessed sensitivity to individual observations. For each contrast, one observation at a time was omitted from either group only when at least two observations would remain. Any shared-control omission was propagated to all dependent contrasts, and Holm correction was recalculated over the original family. This diagnostic examines low-sample sensitivity, not an alternative basis for selecting significant results. Unadjusted 95% confidence intervals for geometric mean ratios are provided as descriptive intervals and are not simultaneous confidence intervals (Table S2, supplied as a CSV file).

## 3. Results

### 3.1. Basal Transcript Abundance Differed Among Anatomical Samples

**Figure 1:**
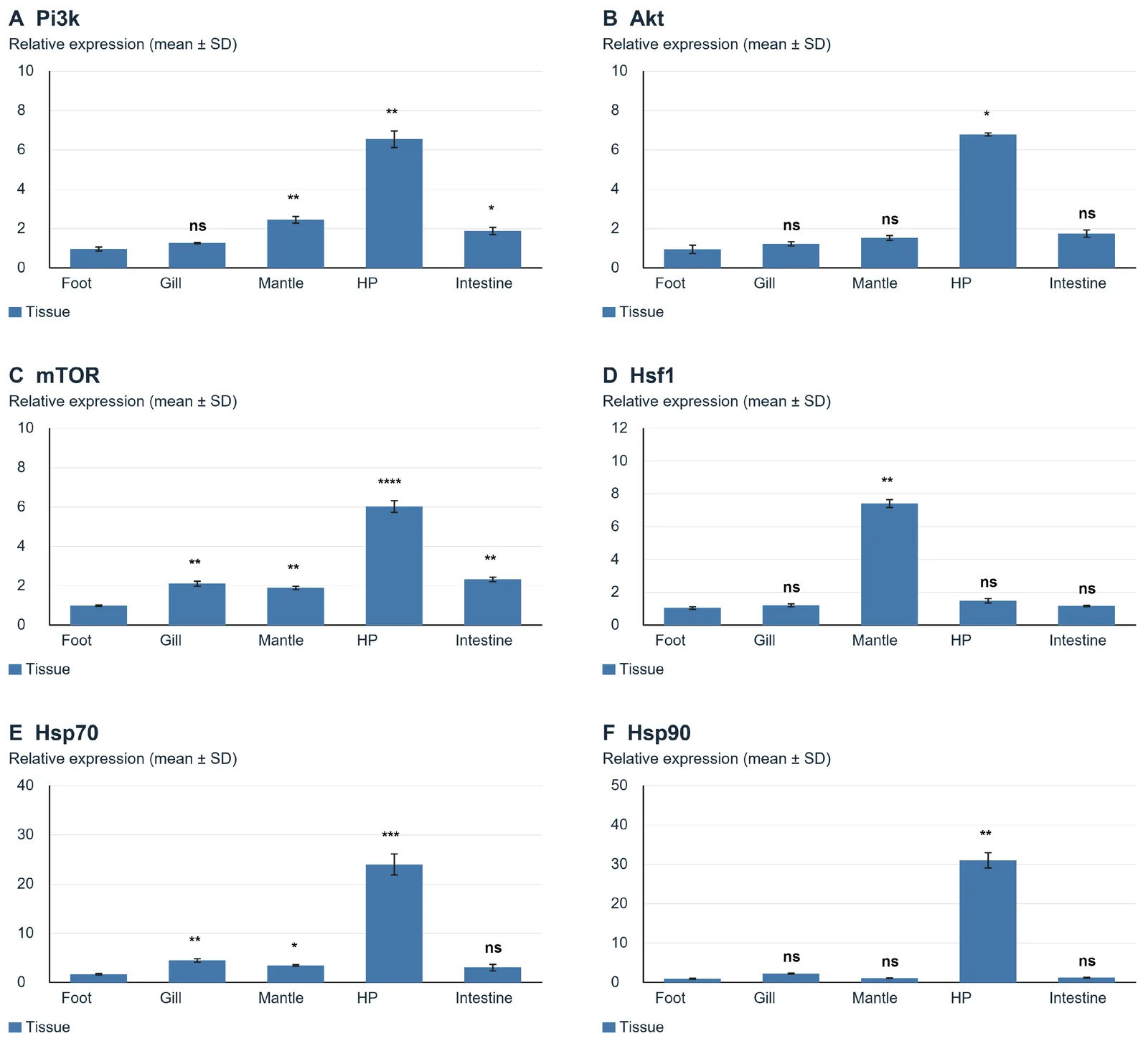
Basal expression of six stress-associated transcripts across five anatomical samples. Panels (**A**–**F**) show *Pi3k*, *Akt*, *mTOR*, *Hsf1*, *Hsp70*, and *Hsp90*, respectively. Bars show arithmetic mean ± SD (*n* = 3 independent biological replicates per group). Each tissue was compared with foot muscle using two-sided unpaired Welch tests on log2-transformed expression, with Holm correction across 24 comparisons. Adjusted *p*-values: * *p* < 0.05, ** *p* < 0.01, *** *p* < 0.001, and **** *p* < 0.0001; ns, not significant.

The six transcripts exhibited different basal tissue profiles (Figure 1). *Pi3k*, *Akt*, and *mTOR* had their highest arithmetic mean relative transcript abundance in the hepatopancreas. Compared with foot muscle, hepatopancreatic geometric mean expression was 6.92-fold higher for *Pi3k* (adjusted *p* = 0.0011), 7.34-fold higher for *Akt* (adjusted *p* = 0.0492), and 6.19-fold higher for *mTOR* (adjusted *p* < 0.0001). These comparisons identify hepatopancreatic enrichment relative to foot muscle; they do not establish significant differences between every pair of tissues.

The heat-shock-associated genes did not share a uniform distribution. *Hsf1* reached its highest mean in the mantle, where its geometric mean was 7.19-fold that in foot muscle (adjusted *p* = 0.0018). *Hsp70* and *Hsp90* instead showed their highest relative transcript means in the hepatopancreas, with corresponding foot-muscle ratios of 14.72 and 34.36 (adjusted *p* = 0.00014 and 0.0020). *Hsp70* was also significantly elevated in the gill and mantle relative to foot muscle. Thus, the basal distribution of *Hsf1* differed from that of the two chaperone transcripts.

### 3.2. The Heat-Selected Population Showed Selective Basal Transcript Differences

**Figure 2:**
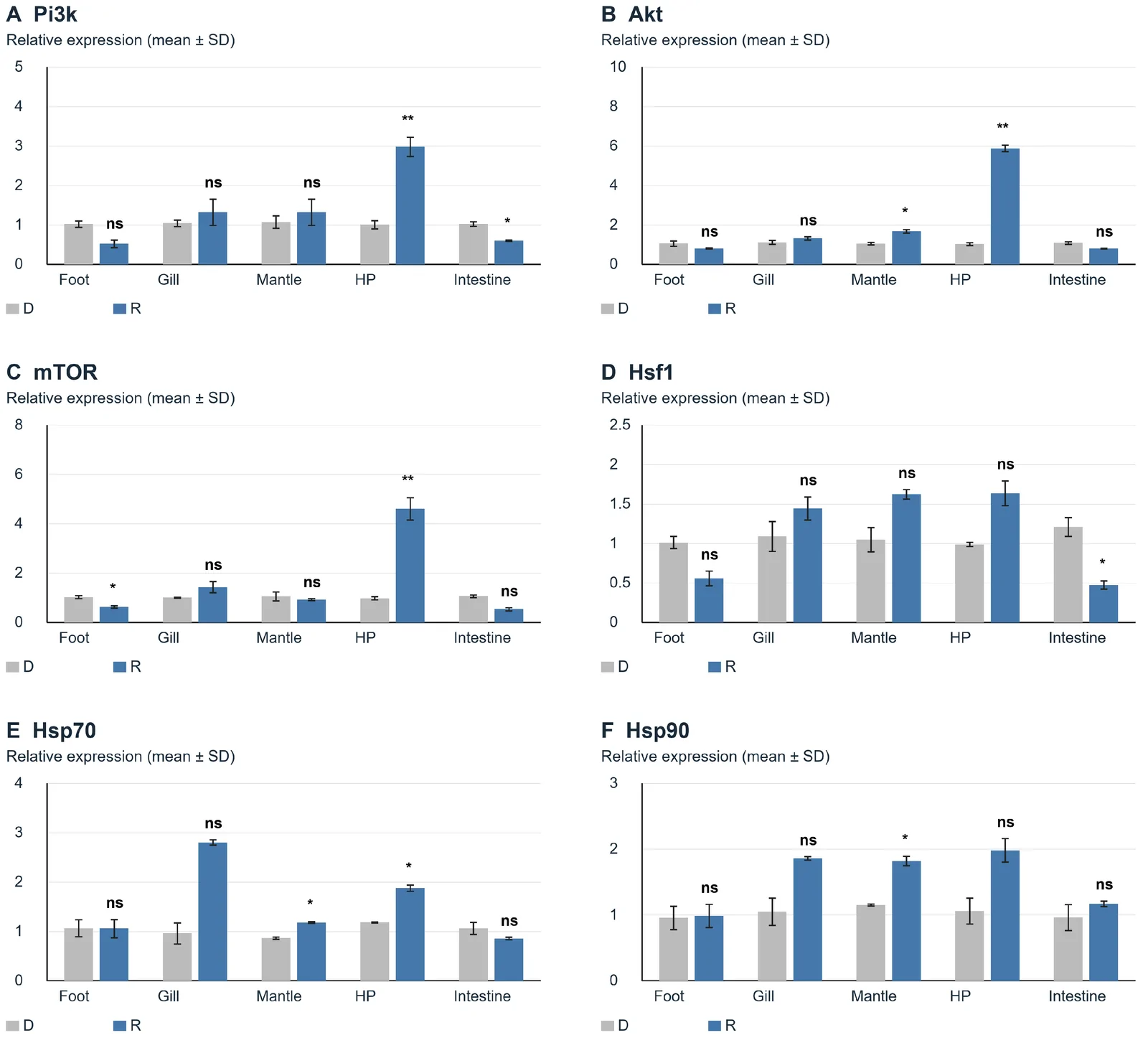
Basal relative transcript abundance in heat-selected (R) and common (D) populations. Panels (**A**–**F**) show *Pi3k*, *Akt*, *mTOR*, *Hsf1*, *Hsp70*, and *Hsp90*, respectively. Bars show arithmetic mean ± SD; *n* = 3 independent biological replicates, except R intestinal *Hsp70* and R gill and intestinal *Hsp90* (*n* = 2). R and D were compared within each tissue by two-sided unpaired Welch tests on log2-transformed expression, with Holm correction across 30 comparisons. Adjusted *p*-values: * *p* < 0.05, ** *p* < 0.01; ns, not significant.

Population differences depended on both tissue and gene (Figure 2). In the hepatopancreas, R had higher *Pi3k*, *Akt*, *mTOR*, and *Hsp70* expression than D, with geometric mean ratios of 2.98, 5.72, 4.72, and 1.59, respectively (all adjusted *p* < 0.05). Hepatopancreatic *Hsf1* and *Hsp90* did not meet the significance threshold after correction. In the mantle, *Akt*, *Hsp70*, and *Hsp90* were higher in R, with ratios of 1.60, 1.37, and 1.58, respectively (all adjusted *p* < 0.05).

The direction of the R/D contrast was reversed for several comparisons. Intestinal *Pi3k* and *Hsf1* were lower in R, with ratios of 0.59 and 0.39, whereas foot-muscle *mTOR* had a ratio of 0.61 (all adjusted *p* < 0.05). No gill contrast reached significance after Holm correction. These within-site contrasts describe a selective pattern; they do not constitute a test of a population-by-anatomical-site interaction or evidence of enhanced thermal resistance.

### 3.3. Adult Relative Transcript Abundance Varied Across Sampled Exposure Times

**Figure 3:**
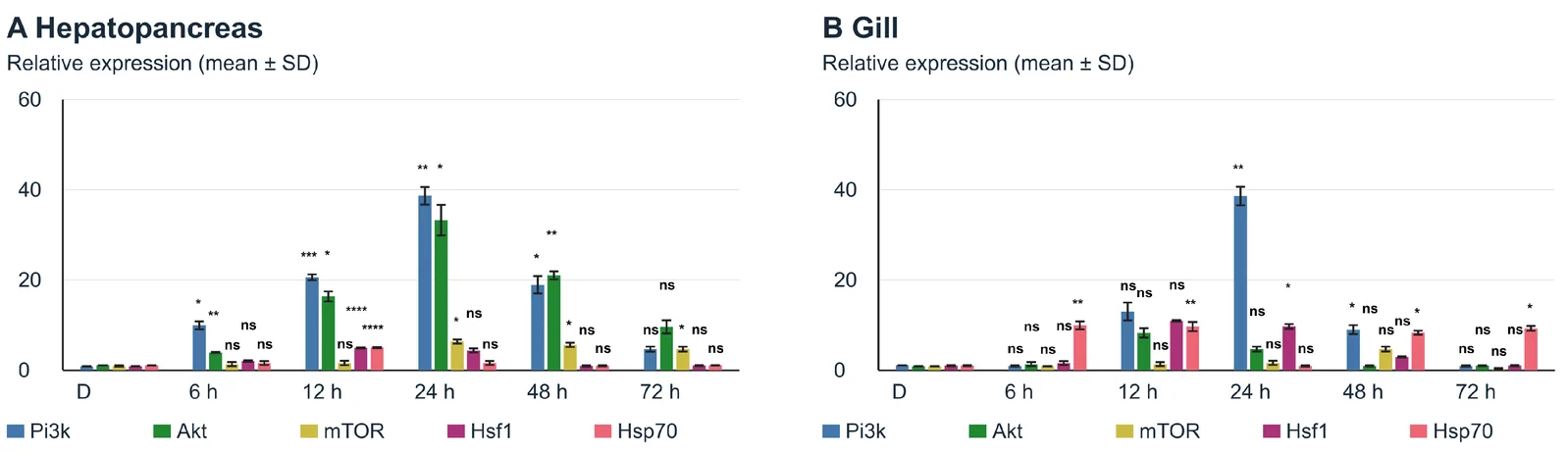
Exploratory adult transcript profiles during exposure to 30 °C. (**A**) Hepatopancreas and (**B**) gill. D denotes the 0 h baseline sampled from the 20 °C control tank; other samples were collected from the heated tank at 6, 12, 24, 48, and 72 h after reaching 30 °C. Values are arithmetic mean ± SD of animal subsamples (*n* = 3, except gill *mTOR* at baseline and 6 h, *n* = 2). Each condition used one tank, so *n* is not the number of independent temperature-treatment replicates. Welch tests on log2 values compared each time with baseline, with Holm adjustment across 125 comparisons in Figure 3 and Figure 4. Stars describe these exploratory sample contrasts, not replicated treatment effects: * *p* < 0.05, ** *p* < 0.01, *** *p* < 0.001, and **** *p* < 0.0001; ns, not significant.

In the common population, hepatopancreatic *Hsf1* and *Hsp70* had their highest sampled arithmetic means at 12 h, with geometric mean ratios of 5.54 and 4.53 relative to baseline (both adjusted *p* < 0.0001; Figure 3A). Their later means were numerically lower. *Pi3k* and *Akt* had their highest sampled means at 24 h, with ratios of 44.05 and 29.58 (adjusted *p* = 0.0013 and 0.0180), and both were elevated relative to baseline at 6–48 h. *mTOR* was elevated relative to baseline at 24, 48, and 72 h. The large *Pi3k*/*Akt* ratios were recalculated from the recorded relative-expression replicates; they were not independently verified against raw Ct or ΔCt. Thus, their scale remains dependent on the original normalization assumptions. The different observed maxima do not establish an earlier biological onset or a statistically different peak time.

Gill *Pi3k* was higher than baseline at 24 and 48 h, and *Hsf1* was higher at 24 h (Figure 3B). *Hsp70* increased at 6 and 12 h and again at 48 and 72 h; the 24 h contrast did not reach significance. Gill *Akt* and *mTOR* varied numerically without significant baseline contrasts after multiplicity correction. This describes the gill contrasts separately and does not establish an organ-by-time interaction.

**Figure 4:**
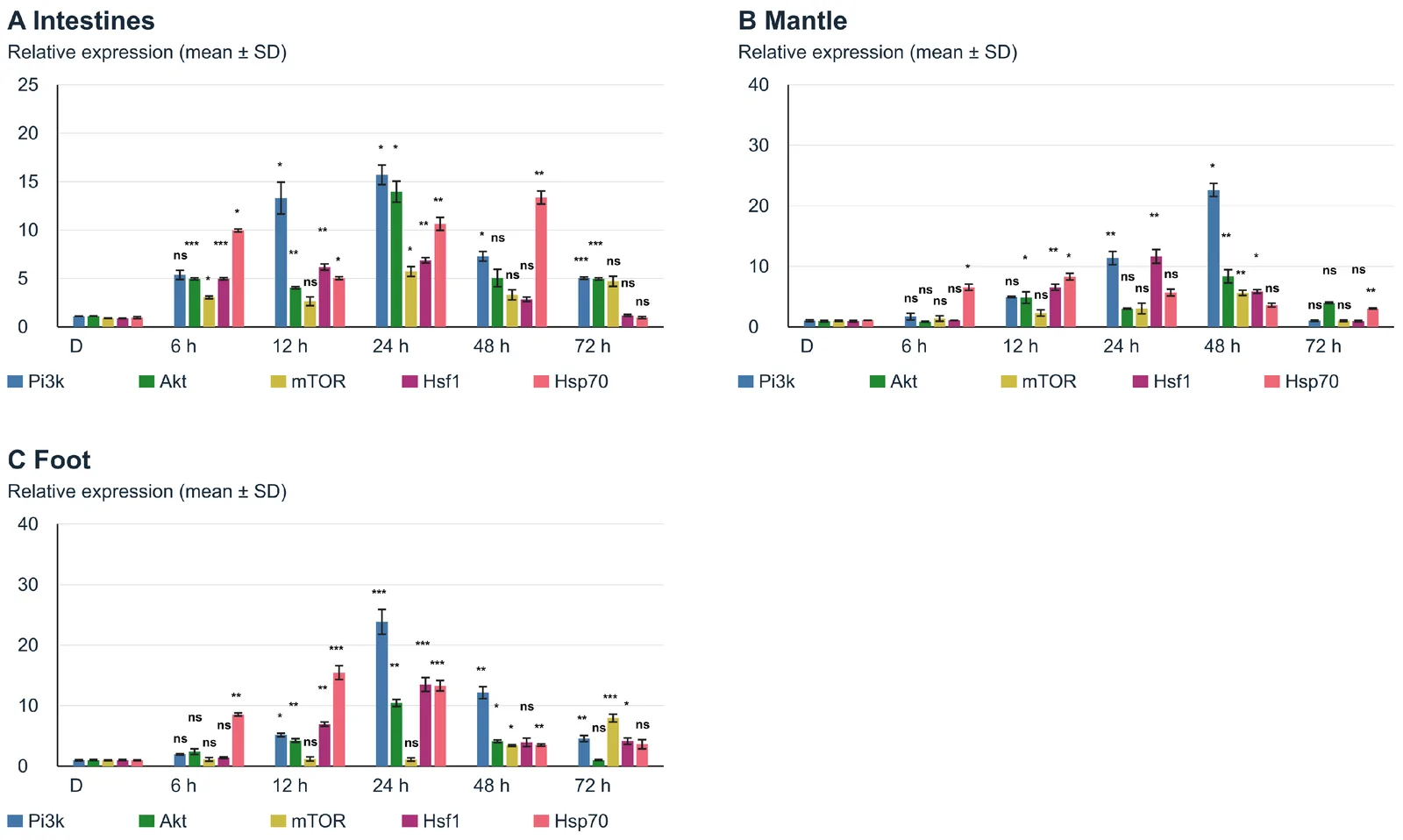
Exploratory adult transcript profiles in (**A**) intestine, (**B**) mantle, and (**C**) foot muscle. D denotes the 20 °C control-tank baseline; 6, 12, 24, 48, and 72 h samples came from the single heated tank after it reached 30 °C. Values are arithmetic mean ± SD (*n* = 3 animal subsamples per group; one tank per condition). Welch tests on log2 values compared each time with baseline, with Holm adjustment across the 125 comparisons in Figure 3 and Figure 4. Labels describe exploratory sample contrasts and do not establish replicated temperature effects: * *p* < 0.05, ** *p* < 0.01, and *** *p* < 0.001; ns, not significant.

Intestine, mantle, and foot-muscle profiles are shown in Figure 4. Mantle *Pi3k* had its highest sampled mean at 48 h, and foot-muscle *mTOR* was elevated relative to baseline at 48 and 72 h. These observations identify sampling intervals for further study. All adult comparisons used the 20 °C control-tank baseline, not successive time points or time-matched unheated samples. Because there was one tank per condition, these are exploratory sample contrasts and cannot distinguish temperature effects from tank and elapsed-time effects.

### 3.4. Within-Stage Transcript Contrasts Following a Brief Developmental Heat Shock

**Figure 5:**
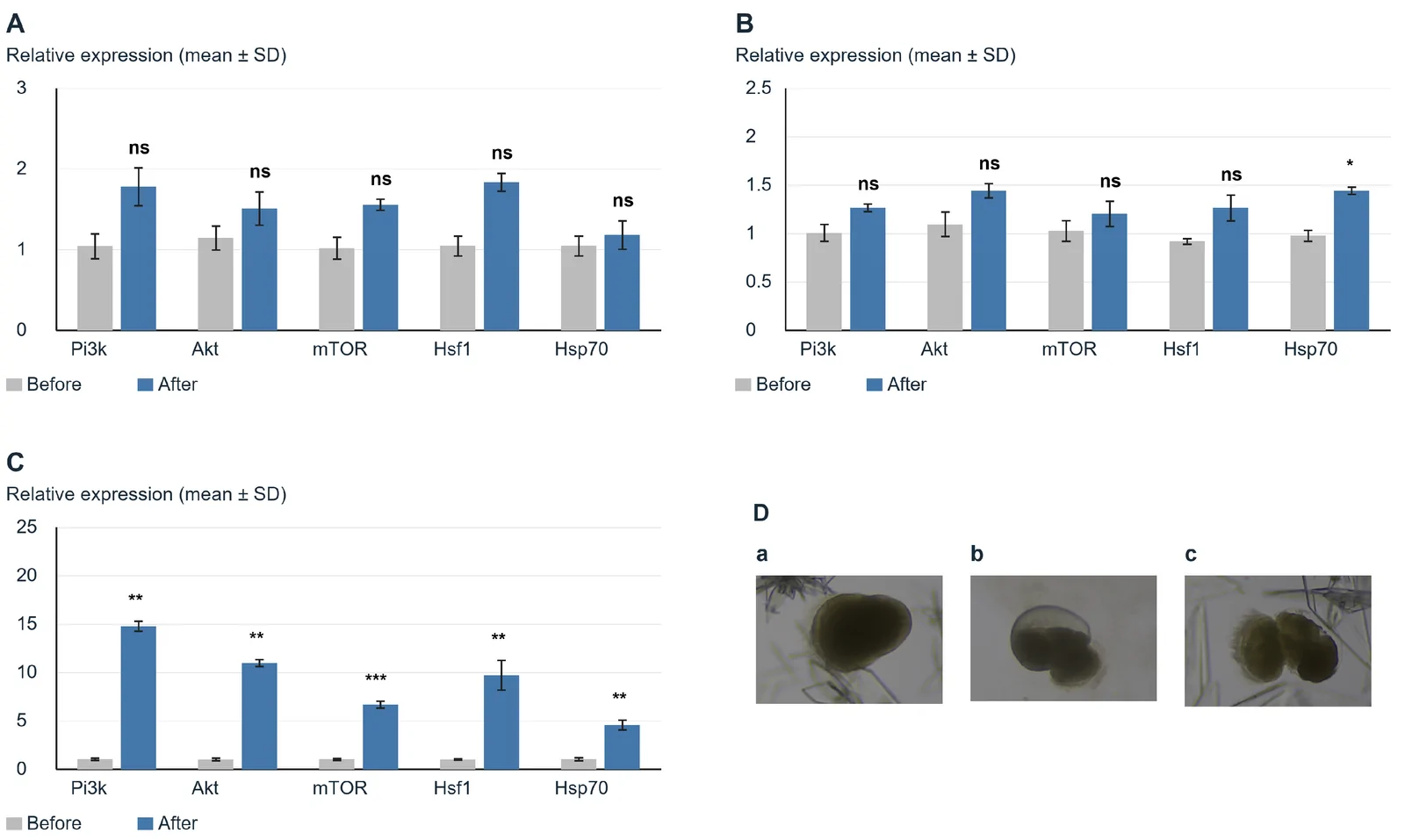
Developmental transcript contrasts after a 15 min heat shock at 30 °C. (**A**–**C**) Trochophore, veliger, and late veliger, respectively; (**D**) Representative developmental images: (**Da**) trochophore, (**Db**) veliger, and (**Dc**) late veliger. Bars show arithmetic mean ± SD of three pools per condition and stage, with 500 larvae per pool. Pools are measurement replicates; independent spawning-batch or culture replication was not specified. After- and before-shock samples were compared by unpaired Welch tests on log2 values, with Holm adjustment across 15 comparisons: * *p* < 0.05, ** *p* < 0.01, and *** *p* < 0.001; ns, not significant. Comparisons describe the sampled pools within each stage and do not test a stage-by-treatment interaction.

After 15 min at 30 °C, none of the five within-stage contrasts reached significance in trochophores (Figure 5A). In veligers, *Hsp70* increased 1.48-fold (adjusted *p* = 0.0243), while the other contrasts did not reach significance (Figure 5B). Within late-veliger samples, *Pi3k*, *Akt*, *mTOR*, *Hsf1*, and *Hsp70* increased 14.51-, 10.99-, 6.66-, 9.62-, and 4.48-fold, respectively (all adjusted *p* < 0.01; Figure 5C). The difference in the number of significant contrasts is descriptive; without a stage-by-treatment analysis it does not demonstrate a broader or stronger response in one stage, and it does not rank developmental thermal tolerance. Representative images of trochophore, veliger, and late-veliger larvae are shown in Figure 5Da–Dc, respectively.

### 3.5. Histological Fields Illustrated Local Structural Irregularities

**Figure 6:**
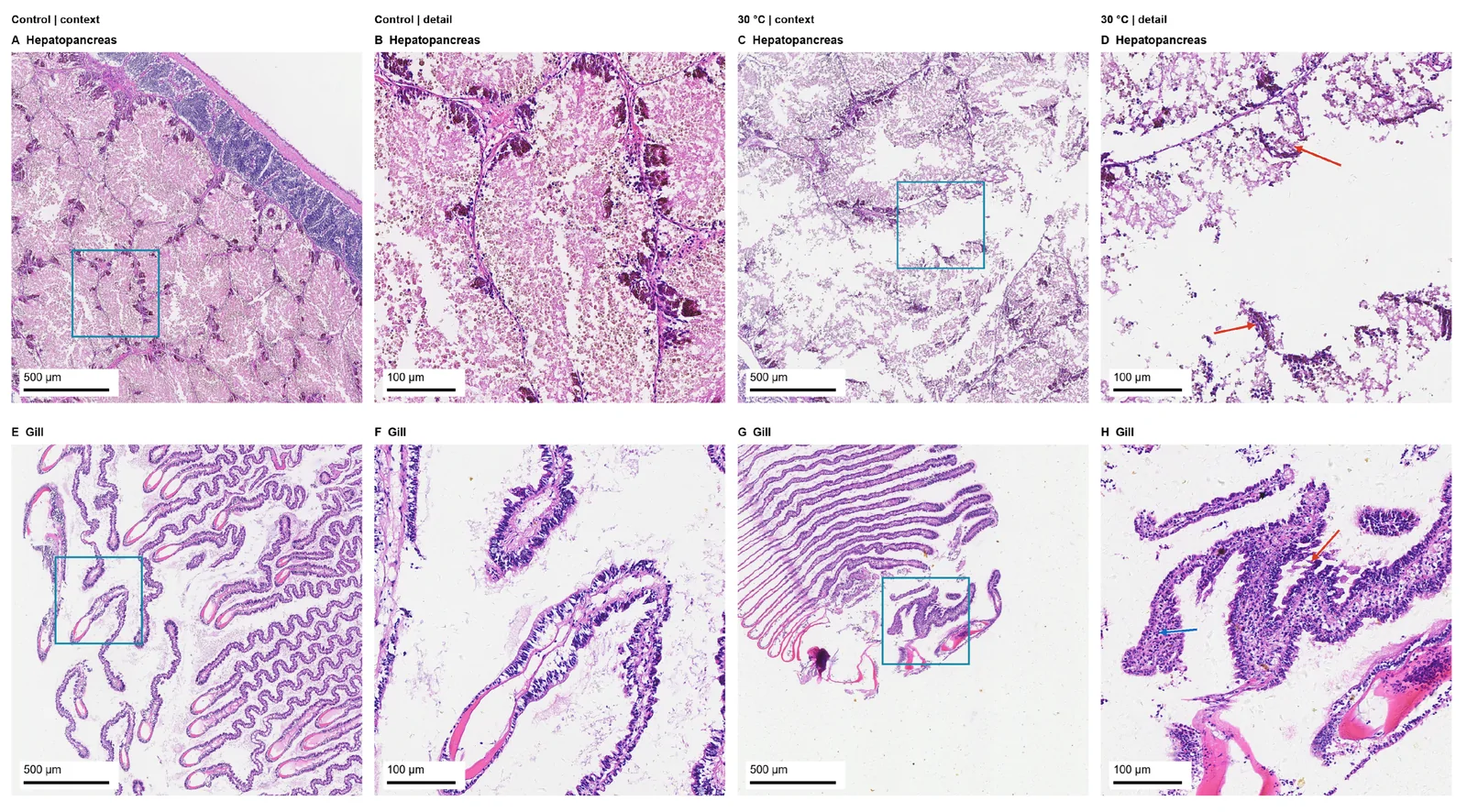
Selected hepatopancreatic and gill fields from control animals at 20 °C and animals after 24 h at 30 °C. (**A**–**D**) Hepatopancreas; (**E**–**H**) gill. (**A**,**B**,**E**,**F**) Controls. (**C**,**D**,**G**,**H**) Heated samples. Cyan boxes locate detail fields. Red arrows indicate focal discontinuities and fragment-like material; the blue arrow indicates a locally dense cellular region. Discontinuities also occur in the control gill. Scale bars: 500 µm (**A**,**C**,**E**,**G**) and 100 µm (**B**,**D**,**F**,**H**). Histology used three animals per condition, ten sections per tissue per animal, and three to five fields per section. The panels derive from one available digitized-slide record per organ and condition and are illustrative fields, not independent replicates or estimates of lesion frequency. Each condition used one tank.

Selected hepatopancreatic fields after 24 h at 30 °C showed discontinuous glandular profiles, fragment-like material, and relatively tissue-poor spaces (Figure 6C,D). Peripheral gill fields contained local discontinuities and dense cellular regions (Figure 6G,H); discontinuities were also present in the control gill (Figure 6E,F). Intestinal and mantle sections provide further morphological context (Figure S2). Additional fields showed discernible glandular boundaries and central gill regions with epithelial continuity (Figure S3). These field descriptions do not estimate lesion prevalence or severity and do not establish a between-condition difference, necrosis, inflammatory-cell identity, or causation by heat.

### 3.6. Exploratory Correlations and Sensitivity Analyses

Temporal group means gave both positive and negative Spearman coefficients (Figure S1). None of the 50 nominal tests remained significant after Holm correction. With six time positions and uncertain exchangeability, this outcome neither demonstrates coordination nor excludes regulatory relationships. No mechanistic conclusions were drawn from these matrices.

Eight contrasts involved an *n* = 2 group, and none reached the corrected significance threshold (Table S2). Of the 194 full-data contrasts, 93 reached adjusted *p* < 0.05. All 93 retained their direction after each permitted single-observation omission, but none remained significant under every omission (Table S3). Reducing a group from three observations to two changes variance and degrees-of-freedom estimates substantially; the original significance calls should therefore be interpreted cautiously rather than described as robust. The recalculated 24 h hepatopancreatic *Pi3k* and *Akt* ratios are detailed in Table S4; these calculations verify relative-quantity arithmetic, not the original Ct normalization.

## 4. Discussion

### 4.1. Basal Population Contrasts and Their Physiological Context

The population comparison identified higher hepatopancreatic *Pi3k*, *Akt*, *mTOR*, and *Hsp70* transcript abundance in heat-selected animals. Together with different contrasts in mantle, intestine, and foot muscle, this supports a nonuniform pattern across the sampled organs/tissues. A hepatopancreatic shift could reflect differences in metabolic allocation or protein maintenance, but neither process was measured directly. The result therefore describes basal transcriptional state rather than constitutive activation of a protective pathway.

Population studies provide useful context without establishing the meaning of these differences. Yao et al. reported population-dependent *Hsp70* and *Hsp90* responses after thermal challenge and divergent growth in southern culture conditions [5]. Chen et al. associated differences in cardiac thermal performance with distinct transcriptomic responses [6]. Yu et al. linked warm-environment domestication to metabolic regulation and energy redistribution [7]. Together, these studies suggest that thermal performance can involve a coordinated physiological phenotype rather than a single universal marker. The present basal comparison adds candidate tissue–gene combinations to that framework, but it does not measure their predictive value for subsequent survival. Acclimation history can also alter the phenotype within a species: juvenile Pacific abalone conditioned at different temperatures showed contrasting survival and metabolic responses to subsequent heating [24]. In European abalone, repeated acute heat exposure improved upper thermal tolerance with a different condition response from chronic warming [25]. Such plasticity means that constitutive expression differences need to be interpreted alongside prior environmental exposure.

Constitutive preparedness remains one possible explanation for higher basal transcript levels, not a demonstrated adaptive function. The two populations underwent the same two-week acclimation, standardizing recent temperature, feeding, and water conditions. Nevertheless, selection history remains confounded with geographical origin and potentially with longer-term husbandry, genetic background, nutritional or reproductive state, and cell composition. Epigenetic differences between geographical populations illustrate further alternatives [8]. The common acclimation and basal transcript comparisons do not establish inherited selection effects or demonstrate that R animals respond earlier, expend less energy, or survive better. The candidate indicators require validation against independently measured tolerance, as distinct from genetic associations with an established thermal phenotype [9].

### 4.2. Organ-Level Transcript Distributions and Exposure Profiles

The hepatopancreas had high basal expression of *Pi3k*, *Akt*, *mTOR*, *Hsp70*, and *Hsp90* within their respective tissue profiles, whereas *Hsf1* was most abundant in the mantle. High hepatopancreatic expression is compatible with the organ’s metabolic and protein-processing demands, but those functions were not measured directly. Physiological work in Pacific abalone demonstrates that digestive-gland enzyme activity changes rapidly under thermal perturbation [1], providing a reason to examine this tissue alongside externally exposed organs. The relative organ-distribution profile of *Hsf1* differed from that of *Hsp70* and *Hsp90*. A comparable hepatopancreatic enrichment of *HSP70* was reported in *Haliotis diversicolor*, where both bacterial and thermal challenges induced expression [26]. This supports its relevance to stress physiology but also illustrates why high *HSP70* expression is not specific to thermal tolerance. Pacific abalone additionally expresses a stress-responsive small heat-shock protein, sHsp19, indicating that the six transcripts measured here represent only part of the chaperone response [27].

The adult data place the highest sampled hepatopancreatic *Hsf1*/*Hsp70* means at 12 h and *Pi3k*/*Akt* means at 24 h. These are descriptive maxima among discrete observations; no test compared peak times or consecutive samples. The profiles can guide denser temporal sampling, but they do not support sequential signaling, an earlier onset of one response, or independence from pre-existing PI3K/AKT proteins. Unobserved maxima between sampling points remain possible. The size of the reported *Pi3k*/*Akt* ratios also depends on single-reference-gene normalization and should be re-evaluated when raw Ct and assay-efficiency information are available. The single heated tank prevents separating these profiles from tank-specific influences.

The 30 °C adult challenge was used to examine expression under a high thermal load. Prior cardiac measurements in other Pacific abalone lines reported Arrhenius breakpoints spanning approximately 28.5–31.9 °C [6], placing 30 °C within a physiologically challenging range in that literature. These values do not establish a transition, sublethal, or lethal threshold for the present stocks or larvae. The adult 6–72 h sampling covers sustained exposure rather than only the initial response; the developmental 15 min treatment addresses a distinct acute-exposure question. No tolerance threshold or optimal challenge duration was determined here.

The gill presented a further distinction: *Hsp70* showed a non-monotonic response, and the *Akt* and *mTOR* baseline contrasts did not reach the adjusted significance threshold. Prior Pacific abalone work has shown that heat-shock-gene responses depend on tissue [11], while *Hsp70* expression can rise and subsequently return toward basal levels during recovery [12]. Sustained heating and post-shock recovery are different exposure designs, so matching a peak time across studies would not establish equivalent physiology. The results instead support considering exposure duration, tissue, and response phase when comparing expression-based stress indicators. The baseline-only contrasts and single tank per condition prevent separating heating from elapsed time and tank effects.

The anatomical profiles suggest complementary targets for physiological follow-up. The hepatopancreatic contrasts are relevant to digestive and metabolic demands, while gill profiles may reflect processes associated with exchange surfaces and environmental exposure. Differences among bulk samples can also reflect their cell composition. Pairing these transcript measurements with organ-specific metabolic or functional endpoints would help distinguish compensatory responses from disturbances in protein homeostasis. The current study measured neither those functions nor translation rates, so transcript differences alone cannot determine whether a response was protective.

### 4.3. Protein Homeostasis and Translation Cannot Be Inferred from Transcript Covariance

Functional studies in other mollusks support the biological relevance of the selected genes. *Hsf1* knockdown affects multiple heat-responsive transcripts in small abalone [14], and HSF1 isoform and promoter differences influence heat-shock responses in oysters [15]. PI3K/AKT-related processes also participate in the small-abalone response to combined environmental stress [16], while oyster experiments have demonstrated regulation of heat-induced caspase activation through phosphorylation [17]. These findings help explain why signaling-associated and heat-shock transcripts are useful to examine together. They do not validate the same regulatory connections in the present samples.

The correlation matrices cannot settle whether these genes are functionally coordinated. Six temporally ordered group means provide limited information and do not supply six independent experimental units. Common exposure can generate similar trajectories without direct interaction, while protein-level interactions may occur despite dissimilar transcript trajectories. Chaperone feedback and phosphorylation can regulate HSF1 independently of *Hsf1* transcript abundance [13,18]. The yeast Hsf1–Hsp70 feedback circuit [28] and human-protein/mammalian-cell evidence for Hsp70-mediated HSF1 trimer disassembly [29] offer mechanistic hypotheses; differences in species, cellular context, and experimental perturbation prevent direct transfer of those mechanisms to the current abalone samples.

The inclusion of *mTOR* also raises a translational question that messenger RNA measurements alone cannot answer. In mammalian cells, mTORC1 regulates protein synthesis and translation of particular messenger RNAs through the 4E-BP–eIF4E machinery [30]. *mTOR* transcript abundance was included to describe a potential link between metabolic signaling, translational demand, and the protein-maintenance functions represented by heat-shock transcripts. In cultured human cells, mTOR perturbation also affected HSF1 phosphorylation and HSP induction [18]. These provide comparative hypotheses, but neither mechanism was tested in the present abalone. Higher *mTOR* transcript abundance cannot be equated with increased kinase activity, overall translation rate, or a favorable balance between protein synthesis and folding capacity. Measurements of pathway-protein phosphorylation, protein-synthesis rates, and chaperone abundance or activity are needed to test those possibilities.

### 4.4. Developmental Transcript Responses Require Stage-Specific Validation

All five within-stage contrasts increased in late-veliger samples, whereas fewer contrasts reached significance in earlier-stage samples. This does not statistically establish a developmental difference in inducibility: stage-by-treatment interactions were not estimated, and a significant result in one stage with a nonsignificant result in another is not itself evidence that their effects differ. Developmental changes in cell composition, transcriptional competence, basal expression, or normalization could contribute. Reference-gene testing in Pacific abalone ranked *ACTB* among the less stable candidates across early development [20], making the lack of documented validation for the present samples particularly relevant. Future comparisons should establish reference-gene stability across both stage and treatment, consistent with MIQE guidance [21]. European abalone studies also show that warming can affect swimming, shell development, and settlement differently [31]; those endpoints and regimes cannot be equated with the present brief transcript assay. The adult and larval exposures had different thermal durations and do not support cross-stage rankings of tolerance. Three pools per stage and condition describe within-cohort sampling; the broodstock counts do not establish independent spawning-batch replication.

### 4.5. Qualitative Morphology and Its Relation to Transcript Measurements

Histology provides descriptive organ-level context after 24 h at 30 °C, with three animals examined per condition and multiple sections and fields per animal. Local discontinuities and dense cellular regions coexisted with more continuous neighboring fields, and control gill sections also contained discontinuities. Published hybrid-abalone work links an environmental challenge to glandular and cellular changes [32], but its algal-bloom exposure and diagnostic evidence cannot be transferred to the present images. Section orientation, processing-related separation, and local anatomical composition remain plausible explanations. The displayed fields came from selected digitized-slide records, without blinded lesion scoring or a quantitative summary across animals. Together with the single tank per condition, this precludes attributing a lesion to heating. Morphology was not quantitatively linked to transcript abundance in matched individuals, so the two datasets provide context rather than a causal transcript-to-injury mechanism.

### 4.6. Statistical and Experimental Limits

The small expression groups, including five groups with *n* = 2, leave variance estimates uncertain and make distributional diagnostics uninformative. Holm adjustment addresses multiplicity within the defined comparison families but cannot correct the absence of independent adult tank replication: temperature is confounded with tank, and baseline-only comparisons also confound heating with elapsed time. Larval pool counts likewise do not demonstrate independent spawning or culture replication. Nonsignificant results do not establish an absence of response. Preliminary single-peak melting curves support assay specificity, but they do not validate amplification efficiencies or *β-actin* stability. Replicated tanks and cultures, time-matched control comparisons, validated normalization, and independently measured performance would be needed for treatment-level inference and interaction tests. Protein abundance, phosphorylation, translation, and functional measurements would address mechanisms beyond the present transcript dataset.

## 5. Conclusions

Pacific abalone showed anatomical variation and selective population differences in relative stress-associated transcript abundance after common acclimation. The heat-selected population had higher hepatopancreatic *Pi3k*, *Akt*, *mTOR*, and *Hsp70* levels, while adult temporal profiles and within-stage larval contrasts identified candidate sampling windows for further study. These patterns require evaluation against independently measured thermal performance. One adult tank per condition, no analyzed time-matched adult controls beyond baseline, unverified larval culture independence, small samples, single-reference-gene normalization, absent protein endpoints, and population-history confounding limit inference. Histology from three animals per condition remains qualitative and does not establish heat-induced injury.

## Data Availability

The data supporting the findings of this study are available from the corresponding authors upon reasonable request.

## Supplementary Materials

The following supporting information can be downloaded at https://www.mdpi.com/article/10.3390/biology15201885/s1, Figure S1. Exploratory Spearman correlations among temporal expression profiles. (A) Hepatopancreas; (B) intestine; (C) gill; (D) mantle; (E) foot muscle. Correlations use group means at six time positions, not six individual animals. Holm correction was applied across 50 gene-pair tests. All adjusted tests were nonsignificant (*p* ≥ 0.05). Six time positions are temporally ordered group means; the significance labels depend on an unverified exchangeability assumption and are not confirmatory tests of biological coordination; Figure S2. Intestinal (A–D) and mantle (E–H) sections from control animals at 20 °C (A, B, E, F) and animals after 24 h at 30 °C (C, D, G, H). Cyan boxes locate detail fields. Scale bars: 200 µm (A, C), 500 µm (E, G), and 100 µm (B, D, F, H). Histology used three animals per condition, ten sections per tissue per animal, and three to five fields per section. The selected panels are illustrative fields from available digitized-slide records, not a quantitative summary across animals. Each condition used one tank; Figure S3. Additional fields from (A) control hepatopancreas, (B) hepatopancreas after 24 h at 30 °C, (C) central control gill, and (D) central gill after 24 h at 30 °C. These selected fields illustrate within-section variation and do not constitute additional animal replicates. The histological sampling design was three animals per condition, with nested sections and fields as described in Section 2.5; treatment tanks were not independently replicated. Scale bars: 100 µm; Table S1. Primer sequences used for quantitative PCR (5′–3′); Table S2. Complete two-sided Welch contrasts on log2 relative-expression values, geometric mean ratios, unadjusted 95% confidence intervals, and Holm-adjusted *p* values. Supplied as Table_S2_Welch_contrasts.csv. Confidence intervals are not corrected for multiplicity. Adult contrasts are exploratory animal-sample comparisons from one tank per condition; larval contrasts do not establish independent spawning/culture replication; Table S3. Single-observation omission sensitivity, with propagation through shared controls and retention of original Holm families. Supplied as Table_S3_sensitivity.csv. A reduced group must retain at least two observations; omissions from original *n* = 2 groups were not performed. Where both groups had *n* = 2, no omission was permissible and sensitivity indicators are reported as not assessed. Omission ranges are not confidence intervals; Table S4. Arithmetic verification of hepatopancreatic 24 h *Pi3k* and *Akt* relative quantities. Supplied as Table_S4_relative_quantity_checks.csv. This does not constitute independent validation from raw Ct or ΔCt.
