# Supplementary material for: Constitutive Expression Differences and Thermal Responses of Stress-Associated Transcripts in Pacific Abalone
Source: Biology (Basel). 2026 Oct 19;15(20):1885. doi: 10.3390/biology15201885 (PMC13701634; doi:10.3390/biology15201885)
Supplement: Supplementary file 1 [file biology-15-01885-s001.zip › biology-4613833 supplementary figures and table S1.pdf]

Supplementary Materials

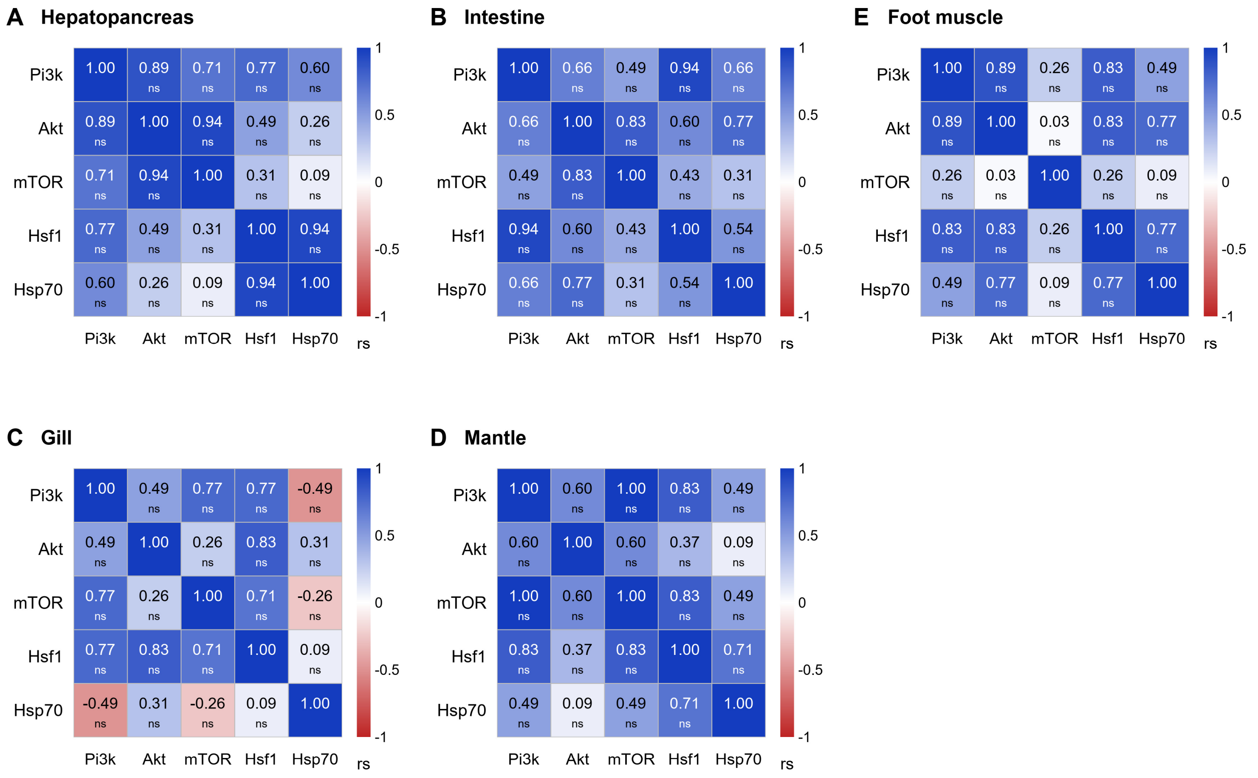

Figure S1. Exploratory Spearman correlations among temporal expression profiles. (A) Hepatopancreas; (B) intestine; (C) gill; (D) mantle; (E) foot muscle. Correlations use group means at six time positions, not six individual animals. Holm correction was applied across 50 gene-pair tests. All adjusted tests were nonsignificant ($p \geq 0.05$). Six time positions are temporally ordered group means; the significance labels depend on an unverified exchangeability assumption and are not confirmatory tests of biological coordination.

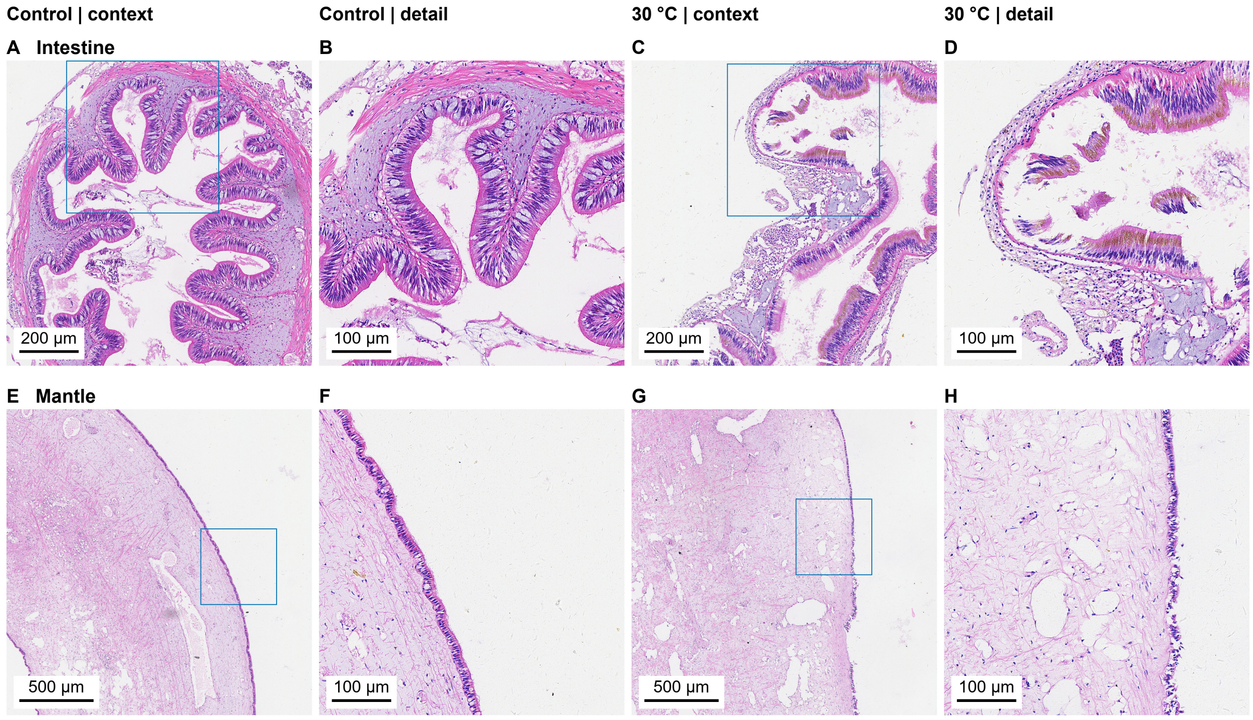

Figure S2. Intestinal (A–D) and mantle (E–H) sections from control animals at 20 °C (A, B, E, F) and animals after 24 h at 30 °C (C, D, G, H). Cyan boxes locate detail fields. Scale bars: 200 µm (A, C), 500 µm (E, G), and 100 µm (B, D, F, H). Histology used three animals per condition, ten sections per tissue per animal, and three to five fields per section. The selected panels are illustrative fields from available digitized-slide records, not a quantitative summary across animals. Each condition used one tank.

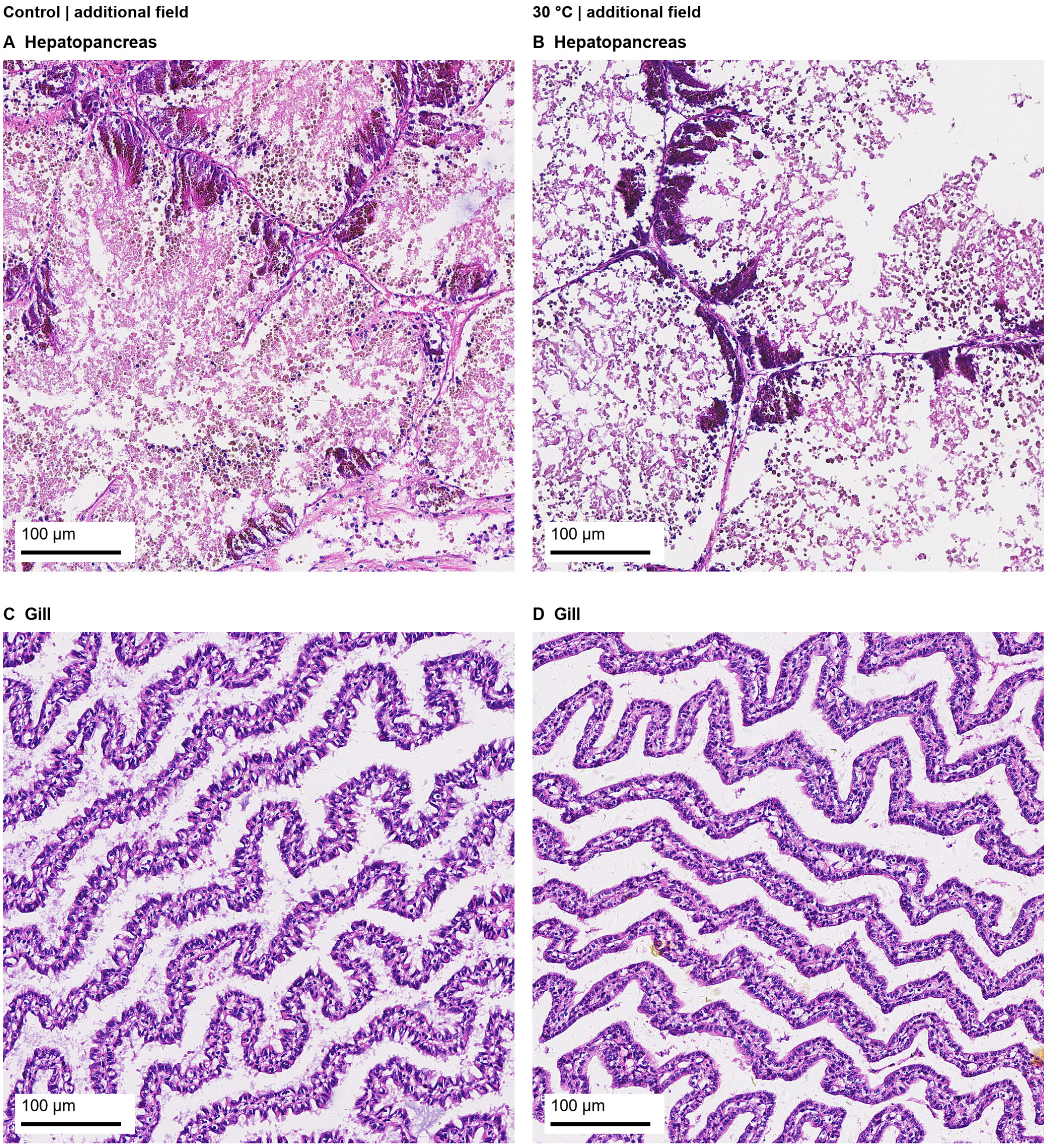

Figure S3. Additional fields from (A) control hepatopancreas, (B) hepatopancreas after 24 h at 30 °C, (C) central control gill, and (D) central gill after 24 h at 30 °C. These selected fields illustrate within-section variation and do not constitute additional animal replicates. The histological sampling design was three animals per condition, with nested sections and fields as described in Section 2.5; treatment tanks were not independently replicated. Scale bars: 100 µm.

Table S1. Primer sequences used for quantitative PCR (5′–3′).

Gene	Forward primer	Reverse primer
Pi3k	AAGCGGAGATGTACCACACG	ACCCATCCTCGGCAATAGAA
Akt	AAGCTGAGAGACCTAAGCCG	CCGTAGATCCGCTTGCATTA
mTOR	TGGAAGGCACTGAGACTGAA	TCCGTACGGTCAATCCTTCT
Hsf1	CAGCAGATTGTCAACAAGCTG	CCGCTGACTGGCATTGTTC
Hsp70	AGGAGGAGATAGAGCGTAT	TCGGTGATGGTCTTCTTG
Hsp90	GGAGAAGGCTGATGCTGACAAGA	CTGTGGCAGATTCCGCTGTAGG
β-actin	CACGGGTATTGTTCTGGACTCTG	ATGAGGTAGTCTGTGAGGTCACGTC

Preliminary assays showed a single sharp melting-curve peak. The sequence table and melting-curve result do not establish numerical amplification efficiencies or experiment-specific β-actin stability.

Table S2. Complete two-sided Welch contrasts on log2 relative-expression values, geometric mean ratios, unadjusted 95% confidence intervals, and Holm-adjusted p values. Supplied as Table_S2_Welch_contrasts.csv. Confidence intervals are not corrected for multiplicity. Adult contrasts are exploratory animal-sample comparisons from one tank per condition; larval contrasts do not establish independent spawning/culture replication.

Table S3. Single-observation omission sensitivity, with propagation through shared controls and retention of original Holm families. Supplied as Table_S3_sensitivity.csv. A reduced group must retain at least two observations; omissions from original n = 2 groups were not performed. Where both groups had n = 2, no omission was permissible and sensitivity indicators are reported as not assessed. Omission ranges are not confidence intervals.

Table S4. Arithmetic verification of hepatopancreatic 24 h *Pi3k* and *Akt* relative quantities. Supplied as Table_S4_relative_quantity_checks.csv. This does not constitute independent validation from raw Ct or ΔCt.
